# Peripheral calcifying cystic odontogenic tumour of the maxillary gingiva

**DOI:** 10.1186/1756-0500-5-455

**Published:** 2012-08-23

**Authors:** Ana Paula de Lima, Dárcio Kitakawa, Janete Dias Almeida, Adriana Aigotti Haberbeck Brandão, Ana Lia Anbinder

**Affiliations:** 1 Department of Bioscience and Oral Diagnosis, School of Dentristy of São José dos Campos, UNESP – Univ Estadual Paulista, Avenida Francisco José Longo, 777. Jd. São Dimas, São José dos Campos CEP, 12245-000, Brazil

**Keywords:** Odontogenic Cyst, Calcifying, Gingival neoplasms, Odontogenic tumors

## Abstract

**Background:**

Odontogenic tumors are lesions that are derived from remnants of the components of the developing tooth germ. The calcifying cystic odontogenic tumor or calcifying odontogenic cyst is a benign cystic neoplasm of odontogenic origin that is characterized by an ameloblastoma-like epithelium and ghost cells. Calcifying cystic odontogenic tumor may be centrally or peripherally located, and its ghost cells may exhibit calcification, as first described by Gorlin in 1962. Most peripheral calcifying cystic odontogenic tumors are located in the anterior gingiva of the mandible or maxilla.

**Case presentation:**

Authors report a rare case of a peripheral calcifying cystic odontogenic tumor of the maxillary gingiva. A 39-year-old male patient presented with a fibrous mass on the attached buccal gingiva of the upper left cuspid teeth. It was 0.7-cm-diameter, painless and it was clinically diagnosed as a peripheral ossifying fibroma. After an excisional biopsy, the diagnosis was peripheric calcifying cystic odontogenic tumor. The patient was monitored for five years following the excision, and no recurrence was detected.

**Conclusions:**

All biopsy material must be sent for histological examination. If the histological examination of gingival lesions with innocuous appearance is not performed, the frequency of peripheral calcifying cystic odontogenic tumor and other peripheral odontogenic tumors may be underestimated.

## Background

A calcifying cystic odontogenic tumor (CCOT) is an extremely rare benign cystic neoplasm that is characterized by an ameloblastoma-like epithelium and ghost cells that have the potential to undergo calcification [1]. Originally, CCOTs were referred to as calcifying odontogenic cysts (COC). The structure was first described by Gorlin in 1962 as a distinct entity and was therefore called Gorlin cyst [2]. COC was considered as a developmental odontogenic cyst in the jaw. In their first report, Gorlin et al. [2] considered this lesion to be a possible analogue of the cutaneous calcifying epithelioma of Malherbe (the pilomatrixoma). COC accounted for approximately 1% of jaw cysts. In 1981, Praetorius et al. [3] studied and reevaluated 16 cases of COC and proposed that the group actually contained two entities, a cyst and a neoplasm. Since then, neoplastic potential has been investigated.

In 2005, the World Health Organization (WHO) designated Gorlin’s cyst as a tumor and described it as belonging to a group of related neoplasms, including the benign cystic-type (CCOT), the benign solid-type dentinogenic ghost cell tumor, and the malignant ghost cell odontogenic carcinoma [1]. The dentinogenic ghost cell tumor seems to be more aggressive than CCOT.

CCOT can occur peripherally or centrally, although only 13% of CCOTs are extraosseous [4]. Extraosseous lesions are typically exophytic masses [5]. In this article, we report an extremely rare case of peripheral CCOT in the maxilla.

## Case presentation

A 39-year-old male patient, without relevant medical history, was referred to the Stomatology Outpatient Clinic of the School of Dentistry of São José dos Campos - UNESP - Univ Estadual Paulista (SP, Brazil) in May of 2007 due to a gingival lesion. A fibrous mass on the attached buccal gingiva of the upper left cuspid teeth was seen during the clinical intraoral examination. The lesion was a 0.7-cm-diameter, painless, firm, sessile nodule of the same color as the adjacent mucosa. The nodule was clinically diagnosed as a peripheral ossifying fibroma. An excisional biopsy was performed under local anesthesia, and the tissue was submitted for histopathological examination.

**Figure 1:**
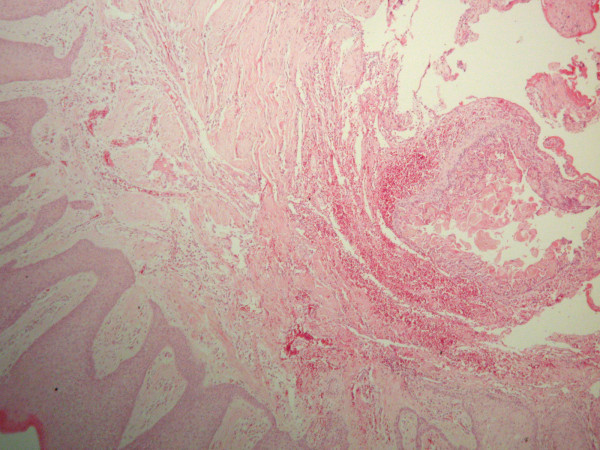
Hematoxylin/eosin-stained section of a well-circumscribed cystic lesion present within the connective tissue (50× original magnification).

**Figure 2:**
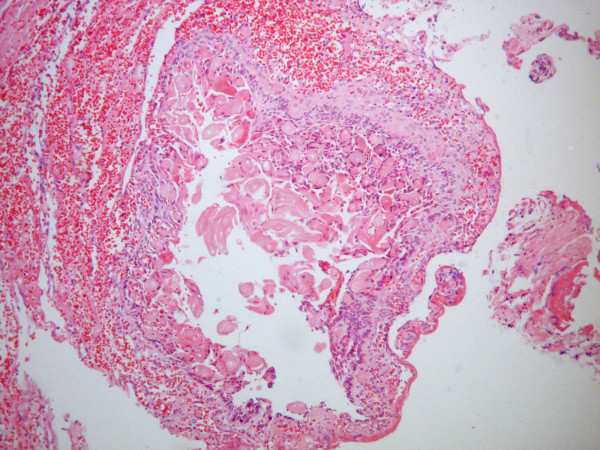
** Hematoxylin/eosin stained section of a cystic lesion lined by ameloblastic-type basal cells.** Note that ghost cells are also evident within an irregular collection of cells that resemble the stellate reticulum (100× original magnification).

Upon microscopic examination, the oral mucosa of the resected sample was found to contain parakeratinized stratified squamous epithelium and underlying fibrous connective tissue. Within the connective tissue, the cystic lesion (Figure 1) was lined with ameloblastic-type basal cells disposed in a palisaded fashion. These cells contained hyperchromatic nuclei that were polarized away from the basement membrane. In addition, eosinophilic ghost cells, a characteristic feature of CCOT, were evident within sheets of loosely arranged cells resembling stellate reticulum (Figure 2). Calcification of ghost cells was also found in the connective tissue wall. Based on these findings, a diagnosis of peripheral CCOT was made.

**Figure 3:**
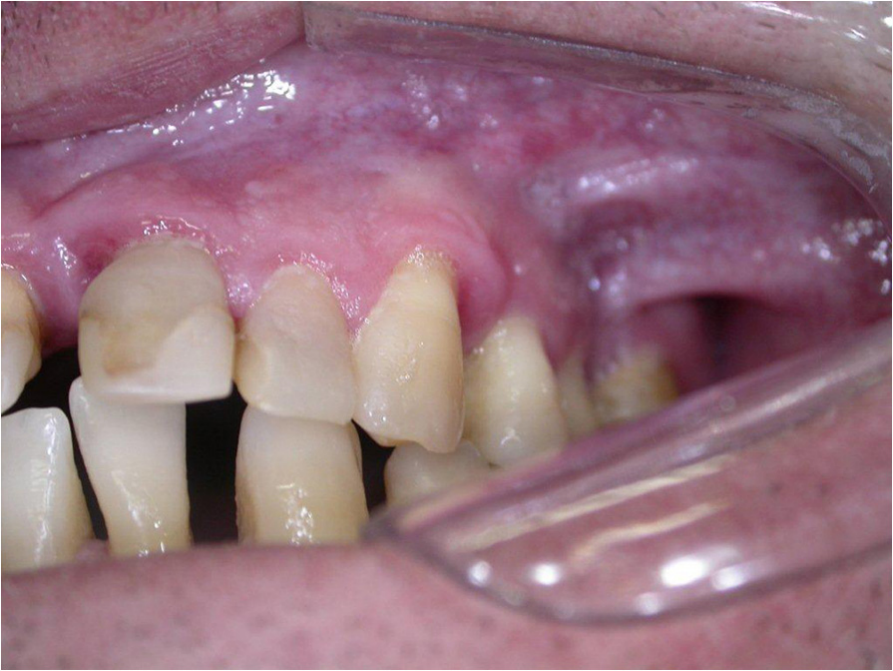
Clinical appearance of the site of the lesion 5 years from the surgery, exhibiting no recurrence.

Following resection of the lesion, the patient’s healing process was uneventful and he was referred for periodontal treatment. After five years’ follow-up, there were no clinical signs of recurrence (Figure 3). The patient has not received periodontal treatment and was referred again.

## Discussion

Odontogenic tumors are relatively uncommon lesions that are derived from the epithelial, mesenchymal, or epithelial/mesenchymal remnants of the components of the developing tooth germ. They are found in the mandible and maxilla and must be considered in differential diagnoses of lesions involving these sites. They can be classified by location as peripheral or central lesions [6].

Peripheral odontogenic tumors are rare and exhibit the histologic features of their central counterpart but occur only in the soft tissue covering the tooth-bearing portion of the maxilla and mandible [7].

Like other neoplasms in the body, odontogenic tumors tend to mimic, at a microscopic level, the cell or tissue of origin. Lesions in this group range from hamartomatous proliferations to malignant neoplasms with metastatic capabilities [6].

CCOT are believed to be derived from odontogenic epithelial remnants within the gingiva or within the mandible or maxilla. The presence of ghost cells, the characteristic microscopic feature of CCOT, may also be seen in other lesions, including ameloblastomas, odontomas, adenomatoid odontogenic tumors, ameloblastic fibroodontomas, and ameloblastic fibromas [6].

Peripheral CCOTs are rare. There were only 45 cases reported in the English-language literature until 1991 [5].

In 2006, Buchner et al. [7] noted that peripheral CCOTs tend to occur more often in mandibular incisor/canine and premolar areas and also occurred more often in females (66.6%) than in males (33.3%). Resende et al. [8], in their review of 44 well-defined cases of peripheral CCOT, also found a slight predilection for females and the anterior region. However, they found a similar distribution throughout the maxillary (40.9%) and mandible (47.7%) regions. In their study, the mean age at the time of diagnosis was 49.4 years.

The peripheral variant of CCOT appears clinically as a well-circumscribed fibrous mass mimicking a nonspecific gingival enlargement. Differential diagnosis of peripheral cases depends on its localization and should include peripheral giant cell lesions, gingival cysts of the adult, fibroma, mucocele, and other benign mesenchymal tumors (e.g., neurofibromas) [9]. Unfortunately, due to the common clinical aspect of the lesion, the case was not photographed.

The discussion about the neoplastic behavior of CCOT is still current. Yoshida et al. [10] studied the immunohistochemical features of 16 cases of intraosseous COC with various histological features, including the proliferative type lining epithelium, that with an ameloblastomatous appearance, and combined odontoma. They found that lining epithelial cells showed cytoplasmic staining for bcl-2 in 12 cases of COCs, but those cells sporadically showed positive reactions for Ki-67 antigen. Immunohistochemical examinations revealed little or no difference in cytodifferentiation or cellular activity among COCs. They concluded that the COC with heterogeneous histological features have neoplastic potential and may not be separate entities within the same histological spectrum.

The nature of ghost cells is not clearly known. Some authors have demonstrated positive expression of amelogenin protein in the cytoplasm of ghost cells, suggesting that epithelium lining CCOT might show ameloblastic differentiation in ghost cells [10].

Due to the non-aggressive behavior of this lesion [8], for most CCOTs, a conservative treatment like enucleation or local resection is appropriate [1]. The lack of recurrence depends on the degree of completion of the excision.

Following enucleation treatment, only a few recurrences have been reported, including intraosseous- and extraosseous-type lesions [1]. In the present case, the patient was monitored for five years following the excision, and no recurrence was detected.

## Conclusions

All biopsy material must be sent for histological examination. If the histological examination of gingival lesions with innocuous appearance is not performed, the frequency of peripheral CCOTs and other peripheral odontogenic tumors may be underestimated.

## Consent

Written informed consent was obtained from the patient for publication of this case report and any accompanying images. A copy of the written consent is available for review by the Editor-in-Chief of this journal.

## Competing interests

The authors declare that they have no competing interests.

## Authors’ contribution

APL, JDA and DK examined the patient. DK carried out the biopsy. APL drafted the manuscript. JDA participated in the design of the manuscript and helped to draft the manuscript. AAHB performed the histological examination. ALA conceived the short report, and participated in its design and coordination. All authors read and approved the final version of the manuscript.
